# Left Inferior Pulmonary Vein–related Reentry Identified Using High-density Activation and Voltage Mapping in Combination with Entrainment Mapping

**DOI:** 10.19102/icrm.2022.130103

**Published:** 2022-01-15

**Authors:** Konstantinos Vlachos, Charis Gkalapis, Michael Efremidis, George Bazoukis, Aggeliki Gouziouta, Pierre Jaïs, Konstantinos P. Letsas

**Affiliations:** ^1^Arrhythmia Unit, Onassis Cardiac Surgery Centre, Athens, Greece; ^2^Department of Electrophysiology-Cardiology, Klinikum Vest, Recklinghausen, Germany; ^3^LIRYC, University of Bordeaux, CHU de Bordeaux, Bordeaux, France; ^4^Service de Rhythmologie, Hôpital Cardiologique du Haut-Lévêque, Pessac, France

**Keywords:** Ablation, atrial fibrillation, mapping, HD Grid

## Abstract

A 61-year-old man with highly symptomatic palpitations presented 13 months after undergoing pulmonary vein isolation for paroxysmal atrial fibrillation. A 12-lead electrocardiogram revealed atrial tachycardia, and the patient was scheduled for mapping, which revealed two regions of reconnection along the posterior part of the region of the left inferior pulmonary vein.

## Case presentation

**Figure 1:**
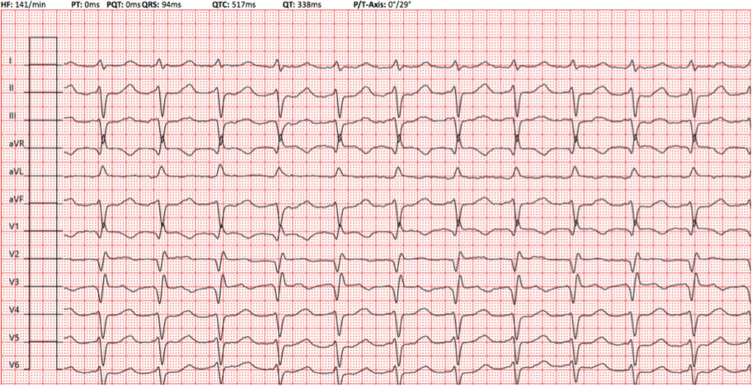
A 12-lead electrocardiogram delineating atrial tachycardia.

**Figure 2:**
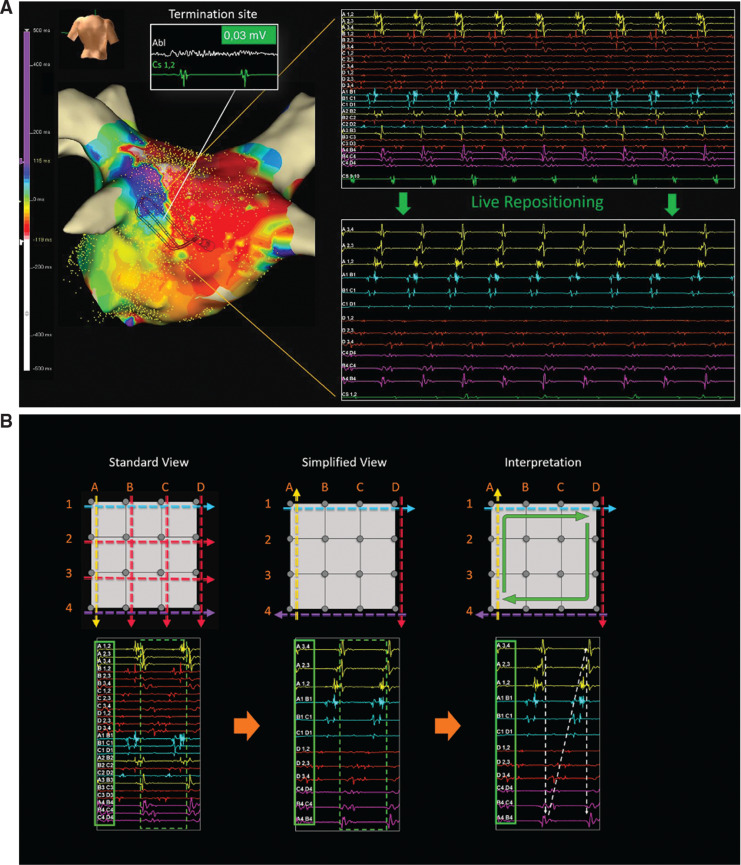
**A:** The Advisor™ HD Grid catheter was used to map the atrial tachycardia (AT). The voltage map revealed two regions of reconnection along the posterior part of the region of the left inferior pulmonary vein (LIPV). The activation mapping of the LIPV gap-related AT in the posterior part of the left atrium (LA) is shown in **Video 1**. Entrainment mapping confirmed the involvement of the posterior part of the LIPV during tachycardia. **B:** The Advisor™ HD Grid Mapping Catheter, Sensor Enabled™ has a paddle shape composed of a 2.5-French 4 × 4 grid (four F1 splines, each with four electrodes) with equidistant 3-mm spacing between the electrodes. The catheter design allows the simultaneous recording of bipolar electrograms in orthogonal directions (along or across the splines), which can be used to visualize two different sets of maps. When a couple of orthogonal bipoles are acquired at the same location, the best duplicate algorithm automatically selects the one with the highest peak-to-peak voltage (HD Wave Solution). Using the standard view, interpretation of registered electrograms is mainly performed from the automated mapping algorithm, resulting in a possible tachycardia circuit at the end of the mapping procedure. Using the simplified view, during mapping acquisition, a continuous flow of information, beat by beat, regarding the tachycardia wavefront direction is made available to the operator simply by them looking at the recorded electrograms. The presentation of only the outer bipolar one allows a quick interpretation, as in our case showing the localized reentry, live and without needing to map the whole LA. As the electrical wavefront passes through the Advisor™ HD Grid catheter, the whole timeline of activation in one beat will appear as a line and the mechanism of the arrhythmia would be established.

A 61-year-old man presented with highly symptomatic palpitations 13 months following pulmonary vein isolation (PVI) for paroxysmal atrial fibrillation (AF). A 12-lead electrocardiogram revealed atrial tachycardia (AT), and the patient was scheduled for mapping and further ablation in our electrophysiology laboratory **(Figure 1)**. The Advisor™ HD Grid Mapping Catheter, Sensor Enabled™ catheter (Abbott, Chicago, IL, USA) was used to map the AT. The voltage map revealed two regions of reconnection along the posterior part of the region of the left inferior pulmonary vein (LIPV) **(Figure 2)**. The activation mapping of the LIPV gap-related AT in the posterior part of the left atrium (LA) is shown in **Video 1**.

**Video 1:** Activation mapping of the LIPV gap-related AT in the posterior part of the LA.

Entrainment mapping confirmed the involvement of the posterior part of the LIPV during tachycardia. Post-pacing interval minus the total cycle length in the posterior part of the LIPV was <20 ms in the two regions with a distance of >2 cm. Local electrograms recorded from the distal electrodes on the Advisor™ HD Grid catheter at the antrum LIPV–LA junction delineated mid-diastolic potentials, which indicated slow conduction through a critical isthmus. The combination of activation, entrainment, and voltage mapping confirmed the diagnosis of an endocardial gap-related reentry in the posterior part of the LA, close to the LIPV **(Figures 2A and 2B)**. Two seconds of ablation in this low-voltage region (amplitude of 0.03 mV, duration of 83 ms in the bipolar signals) at a power setting of 30 W resulted in termination of the tachycardia. Further ablation was performed along the posterior part of the LIPV–LA junction. During this procedure, 21,000 electrical points were automatically collected over a period of 12 minutes and a procedure time of 150 minutes. No atrial arrhythmia (AF or AT) could be induced at the end of the procedure.

## Discussion

High-resolution mapping of the atria can be rapidly and accurately performed using the Advisor™ HD Grid catheter for the ablation of AF, redo AF ablations, and post-PVI ATs.^1^ In addition to providing rapid voltage and activation maps, this catheter can be positioned in such a way so as to rapidly localize gaps and provide information on the direction of conduction. In particular, omnipolar mapping will allow for accurate directionality as well as a more detailed substrate mapping in the presence of changes in the direction of conduction. The unique shape and predictable electrode location allow for the rapid mapping of connections between the LA and the pulmonary veins as well as the diagnosis of complex post-PVI ATs.^1^

The Advisor™ HD Grid mapping catheter has a paddle shape composed of a 2.5-French 4 × 4 grid (four F1 splines, each with four electrodes) with equidistant 3-mm spacing between the electrodes. The catheter design allows the simultaneous recording of bipolar electrograms in orthogonal directions (along or across the splines), which can be used to visualize two different sets of maps. When a couple of orthogonal bipoles are acquired at the same location, the best duplicate algorithm automatically selects the one with the highest peak-to-peak voltage (HD Wave Solution; Abbott).^2^ We used the Advisor™ HD Grid mapping catheter to create local activation timing maps and voltage maps. Zones with a peak-to-peak bipolar voltage of <0.05 mV and 0.05–0.5 mV were defined as scar ultra–low-voltage zones and low-voltage zone, respectively, via the EnSite Precision™ three-dimensional mapping system (Abbott). Both bipolar (30-Hz high-pass filter, 300-Hz low-pass filter, and noise filter) and unipolar (0.5-Hz high-pass filter, 100-Hz low-pass filter, and noise filter) filters were switched on. The HD Wave Solution algorithm on the EnSite Precision™ mapping system assigns the highest peak-to-peak voltage to anatomical points collected by the Advisor™ HD Grid catheter, thus reducing the possibility of missing viable tissues within the low-voltage areas (“scars”) in the LA. This allows us to identify and delineate circuits and the critical isthmus in AT post-AF ablation. Areas with a voltage 0.5–1.5 mV were defined as abnormal low-voltage areas. However, these thresholds may be dependent on the electrode spacing and the angle between the bipolar direction and the activation direction. Electrograms can vary depending on multiple factors, such as electrode size, interelectrode spacing, and direction of the activation wavefront.^3–5^ Bipolar voltage and electrogram characteristics can be further affected by variations in electrode size and/or interelectrode distance of the mapping catheter, direction of the activation wavefront, and wall thickness. Mapping of scar-related AT can be challenging even with the use of a high-density mapping catheter. The best duplicate algorithm of the EnSite Precision™ mapping system uses orthogonal bipoles to compare signal amplitude for collocated mapping data to more accurately display the highest amplitude signal. The Advisor™ HD Grid catheter has 16 equidistant electrodes, allowing for the assessment of conduction directionality as well as omnipolar electrography. This allows for the differentiation of far-field and near-field signals using a single catheter, which can be helpful for the determination of an endpoint in PVI. The Advisor™ HD Grid is a directional high-density mapping catheter which not only identifies local electrical signal but, more importantly, also captures the direction of wavefront propagation, especially in low-voltage zones. The unique shape and predictable electrode location allow for the rapid mapping of connections between the LA and the pulmonary veins as well as the diagnosis of complex post-PVI ATs. This enhances the accuracy of mapping in atrial arrhythmias. This catheter can also be easily used to assess the bidirectional conduction block along a line of ablation.
